# Improvement of biosurfactant production by *Rhodococcus ruber* IEGM 231 grown on waste cooking oil, their emulsifying activity and antioxidant potential

**DOI:** 10.1186/s40643-026-01102-8

**Published:** 2026-08-10

**Authors:** Maria S. Kuyukina, Anastasiia V. Krivoruchko, Irina B. Ivshina

**Affiliations:** 1 Microbiology and Immunology Department, Perm State University, 15 Bukirev street, Perm, Russia 614068; 2 Institute of Ecology and Genetics of Microorganisms, Perm Federal Research Center, Ural Branch of the Russian Academy of Sciences, 13 Golev street, Perm, Russia 614081

**Keywords:** Biosurfactant, Glycolipid, *Rhodococcus*, Waste cooking oil, Response surface methodology, Emulsification, Antioxidant activity, Mycolyltransferase

## Abstract

In recent years, the production of microbial biosurfactants, increasingly used in various industries and agriculture, from low-cost renewable substrates is intensively studied. Large volumes of food waste, in particular waste cooking oil, are promising substrates for the production of biosurfactants on an industrial scale. However, studies on the ability of *Rhodococcus* bacteria, well-known biosurfactant producers, to grow on this substrate and the functional characteristics of synthesized biosurfactants are still scarce. In this study, the possibility of improving biosurfactant production by *Rhodococcus ruber* IEGM 231 grown on waste cooking oil was investigated using multifactor analysis and response surface methodology (RSM). Using standardized parameters for the concentrations of carbon and nitrogen sources in the fermentation medium, a Pareto diagram was constructed, which shows a direct dependence of the crude biosurfactant yield on the oil concentration, while the concentrations of sugar and inorganic nitrogen salts were found insignificant. Using RSM, the optimal ratio of the medium parameters was determined for the maximum yield of crude biosurfactants: oil (+ 1), sugar (0), NH_4_- and NO_3_-containing salts (− 1) in the following concentrations: 5.0 vol%, 2.5 g/L, 0.1 and 0.2 g/L. Crude biosurfactants (40 g/L) obtained from excess waste oil under nitrogen limitation contained three glycolipid fractions, including predominant monoacyl trehalose, previously identified for this strain grown on *n*-hexadecane, and a considerable amount of residual non-converted acylglycerides. The synthesized biosurfactants produced stable emulsions from petroleum products, food and cosmetic oils and demonstrated high antioxidant capacity in the in vitro ABTS (2,2-azino-*bis*-3-ethylbenzothiazoline-6-sulphonic acid) radical scavenging test. Overexpression of the *fbpB* gene encoding mycolyltransferase, a key enzyme of the final stage of trehalolipid assembly was revealed in the cells grown on waste cooking oil, which indicated the induction of the synthesis of glycolipid biosurfactants. These findings open up the possibility of valorization of large-scale food oil waste into value-added biosurfactants using *Rhodococcus*.

**Graphical abstract:**

**Figure:**
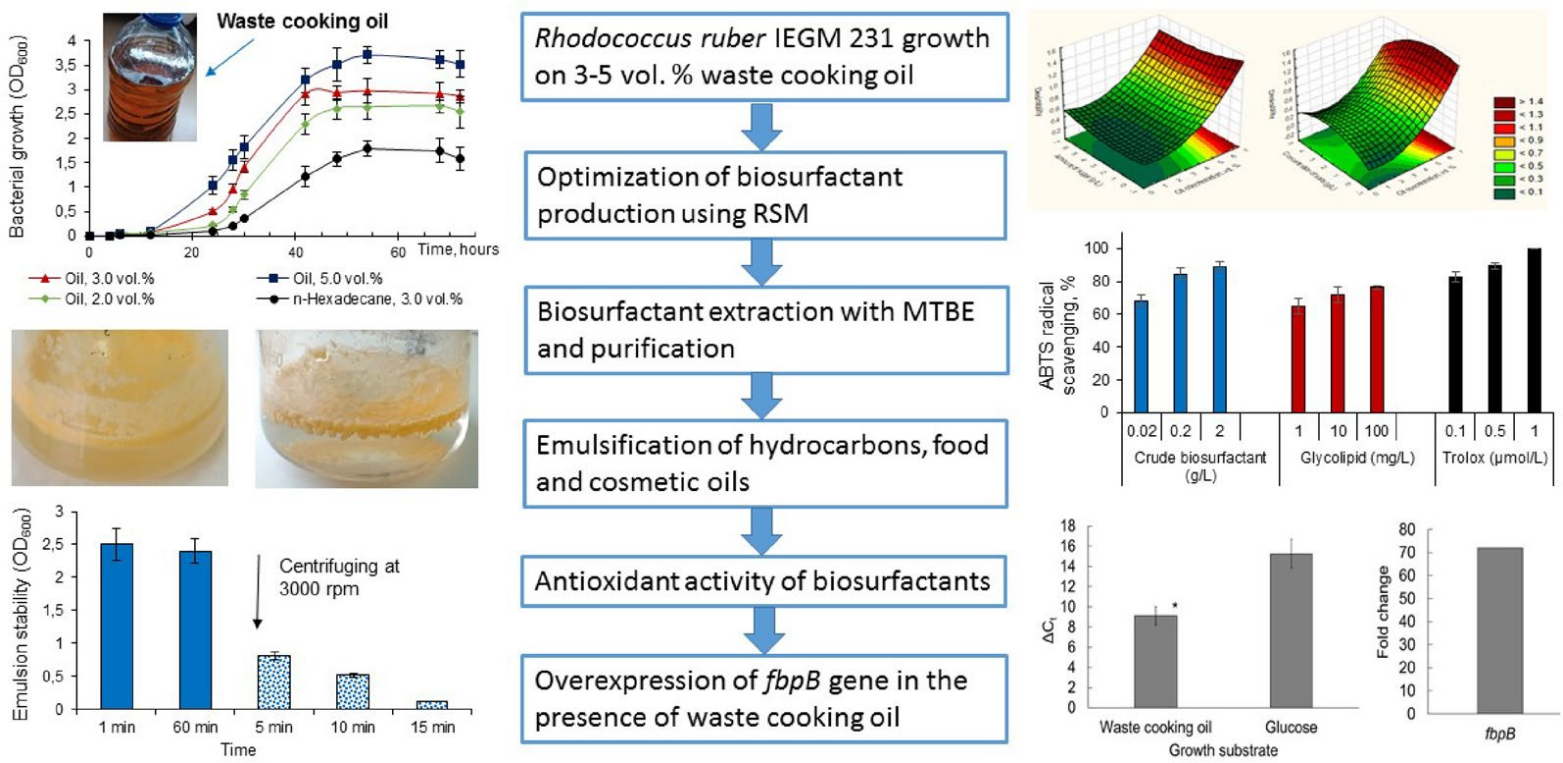

**Supplementary Information:**

The online version contains supplementary material available at 10.1186/s40643-026-01102-8.

## Introduction

Microbial biosurfactants are increasingly used in various industries, agriculture, environmental biotechnologies, medicine, cosmetology, and food processing. However, their massive market promotion is hindered by their relatively low yield and high cost of production compared to chemical surfactants (Christofi and Ivshina 2001; Mukherjee et al. 2006; Thundiparambil Venu et al. 2024). Fermentation substrates or raw materials can contribute up to 89% of the total production cost of biosurfactants, which determines the importance of selecting inexpensive and affordable substrates (Ashby et al. 2013).

Traditionally, microbial biosurfactants were obtained using immiscible substrates such as petroleum hydrocarbons, most often *n*-hexadecane, due to the alkanotrophic ability of producer strains (Franzetti et al. 2010). However, the subsequent use of such products is limited by environmental insecurity due to the possible presence of residual hydrocarbons in crude biosurfactants (Kuyukina et al. 2001). Therefore, it is relevant to search for non-toxic renewable sources of raw materials, for example, oil and fat-containing waste from the food industry, the use of which can serve as an effective bioeconomics strategy (Thundiparambil Venu et al. 2024). The use of waste cooking/vegetable oil for the production of biosurfactants has previously been studied on representatives of yeast and bacteria (mainly *Pseudomonas* species for the synthesis of rhamnolipids) (Chen et al. 2018; Niu et al. 2019; Jiang et al. 2020; Liepins et al. 2021).

Members of the genus *Rhodococcus*, well-known for their diverse biological properties and metabolic versatility, produce biosurfactants in response to the presence of liquid hydrocarbons in the growth medium. These biosurfactants are predominantly cell-bound glycolipids containing trehalose as the carbohydrate. *Rhodococcus* trehalolipids possess a variety of physico-chemical properties and biological activity comparable to other microbiological surfactants, which determines their potential application in industrial and environmental biotechnologies (Franzetti et al. 2010; Kuyukina and Ivshina 2019). Along with *Rhodococcus*, trehalolipids are produced by closely related genera such as *Mycobacterium*, *Nocardia*, *Corynebacterium*, *Gordonia*, *Dietzia*, *Tsukamurella*, *Skermania* and *Williamsia* (Kuyukina and Ivshina 2019). White et al. (2013) evaluated the production of trehalolipids by a marine bacterium *Rhodococcus* sp. PML026 using sunflower oil as a hydrophobic substrate. More recently, Malavenda et al. (2015) isolated several psychrotolerant *Rhodococcus* strains from polar regions, which were able to produce biosurfactants when grown on tetradecane and sunflower oil. However, there are only a few publications on the use of waste cooking oil as a carbon source for *Rhodococcus* and the effect of this substrate on the biosurfactants produced (Pirog et al. 2013; Andreolli et al. 2023). It should be noted that development of a cost-effective technology for biosurfactant production requires fundamental knowledge on the metabolic pathways involved in the glycolipid synthesis from such alternative substrates and coresponding genetic mechanisms.

We previously studied the biosynthesis and bioactive properties of trehalolipid biosurfactants produced by *Rhodococcus ruber* IEGM 231 grown on hydrocarbon substrates (*n*-hexadecane and *n*-dodecane), including immunomodulating, membranotropic and antiadhesive activities (Philp et al. 2002; Kuyukina et al. 2016, 2020). In the present study, the possibility of improving biosurfactant production by this strain grown on waste cooking oil was investigated using response surface methodology. Emulsifying activity of the produced biosurfactants against petroleum hydrocarbons, food and cosmetic oils, as well as their antioxidant potential, was evaluated in order to identify their possible industrial applications. Additionally, the expression of mycolyltransferase gene in the presence of waste cooking oil was studied for the first time to reveal the substrate-dependent regulatory mechanisms of biosurfactant synthesis in *Rhodococcus*. Thus, the present study addressed a critical issue of the valorization of waste cooking oil for biosurfactant production in order to ensure environmental sustainability and carbon footprint reduction.

## Materials and methods

### Bacterial strain and growth conditions

The *Rhodococcus ruber* IEGM 231 strain from the Regional Specialized Collection of Alkanotrophic Microorganisms (acronym IEGM; WDCM 768; www.iegmcol.ru) was used. This strain, previously isolated from spring water in an oilfield in the Perm Region of Russia, uses *n*-hexadecane, propane and *n*-butane as the sole carbon source, degrades organic pollutants, serves as a bioremediation agent for oil-contaminated soil and produces glycolipid biosurfactants with immunomodulatory and antiadhesive properties (http://www.iegmcol.ru/strains/rhodoc/ruber/r_ruber231.html). The mineral RS medium contained (g/L): K_2_HPO_4_–2.0, KH_2_PO_4_–2.0, KNO_3_–1.0, (NH_4_)_2_SO_4_–2.0, NaCl–1.0, MgSO_4_–0.2, CaCl_2_–0.02, and a trace element solution–1 mL/L. Yeast extract (1.0 g/L) was added as a growth factor. Waste cooking oil from a local fast food restaurant was filtered to remove solids and used as a carbon source in concentrations of 2.0, 3.0 and 5.0 vol%. Alternatively, *n*-hexadecane (3.0 vol%) was used as a model hydrocarbon substrate for biosurfactant production. Bacteria were grown in 250 mL Erlenmeyer flasks containing 100 mL of the medium incubated under orbital stirring at 71.5 g and 28 °C for 3 days with an inoculum of 5.0%.

In experiments to optimize the culture medium, due to the large number of experimental variants, refined deodorized sunflower oil “Sloboda” (Belgorod, Russia) in concentrations of 2.0, 3.0 and 5.0 vol% and granulated sugar in concentrations of 1.0, 2.5 and 5.0 g/L were used. The concentration of nitrogen salts (KNO_3_ and (NH_4_)_2_SO_4_) varied in the range of 0.1–1.0 and 0.2–2.0 g/L, respectively.

Bacterial growth on waste cooking oil was monitored both macroscopically and microscopically. Macroscopical analysis to evaluate the growth of the microorganism included turbidimetry (OD_600_) and the visual observation of morphological patterns. Before measuring turbidity, the cultures were vigorously shaken to obtain homogeneous samples. Microscopical analysis to monitor oil emulsification and uptake, possible contamination, biosurfactant production, and microorganism growth was conducted using a Zeiss Axioskop microscope (Carl Zeiss, Germany).

### Biosurfactant extraction and purification

A crude biosurfactant was extracted from the bacterial culture with methyl tert-butyl ether (MTBE) as previously shown (Kuyukina et al. 2001). For this, the upper hydrophobic layer of a bacterial culture was collected and treated with ultrasound at 44 kHz, 0.7 A, and 4 °C for 1.5 min, then it was extracted with MTBE at 2:1 ratio. Extraction was performed on an orbital shaker (71.5 g) at 28 °C for 4 h. After that, the extract was centrifuged at 1500 g for 10 min (Biosan LMC-3000) and the solvent was removed using a Laborota 4000 rotary evaporator (Heidolph, Germany) at a solvent evaporation temperature of 50 °C under reduced pressure. Resulting crude extracts were freeze-dried and stored under nitrogen.

### Determination of the emulsifying activity

The emulsifying activity of crude biosurfactants was determined by a modified test tube method. For this, 0.5 mL of distilled water, 0.5 mL of *n*-hexadecane or other hydrophobic substrate and 0.01 mL of biosurfactant were mixed in microtubes at room temperature in a five-fold repeat. A similar sample without the addition of biosurfactant was used as a control. Microtubes were sonicated (23 kHz, MSE Soniprep 150) for 2 min and then settled for 6 min, 1 h, 24 h and 2 weeks. The type of emulsion formed (oil-in-water or water-in-oil) was determined and the emulsification index was calculated in % as the ratio of the emulsion layer height to the total height of the liquid column in the microtube. The use of microtubes and small volumes of test substances, as well as ultrasonic treatment of emulsions for better homogenization and 5-fold repeatability allowed to increase the reproducibility of the results. The hydrodynamic stability of the formed emulsions was assessed by the change in the optical density (OD_600_) after centrifugation of the emulsion at 1500 g for 5–15 min.

### Qualitative structural analysis of biosurfactants

The qualitative analysis of biosurfactants was carried out by thin-layer chromatography (TLC) after dissolving in chloroform. Following solvent systems were used: (i) chloroform-methanol-water (85:15:2) for glycolipids, (ii) *n*-hexane-chloroform-acetic acid (20:80:0.5) for acylglycerols and fatty acids. Raw and waste vegetable oils were used as controls. The detection of lipids was carried out using spray reagents consisting of 15% sulfuric acid and 10% phosphomolybdic acid in ethanol, heated to 110 °C. Identification of spots was carried out using reference compounds (triacylglycerides. monoacyltrehalose) and Rf values from the literature.

### Isolation of the glycolipid fraction

Biosurfactant fractions were separated using flash chromatography on the Silicagel 60 column (40 × 150 mm; Buchi) with a solvent system of increasing polarity: chloroform: methanol: water in proportions 65:5:0.3 and 65:7.5:0.3 (Gein et al. 2020). Sequentially eluted from the column 10 mL fractions were collected in separate tubes and analyzed using TLC. Fractions with TLC-detectable glycolipids were combined and, after solvent rotary evaporation at 65 °C, the amount of isolated glycolipids was determined gravimetrically.

### Analysis of antioxidant capacity

Colorimetric analysis of total antioxidant capacity (TAOC) of crude biosurfactant and isolated glycolipid fraction was performed in 96-well microplates using TAOC kit (Elabscience, catalog number E-BC-K271-M) according to the manufacturer’s assay protocol. This method assays the relative ability of antioxidants to scavenge the ABTS (2,2-azino-*bis*-3-ethylbenzothiazoline-6-sulphonic acid) radicals generated in the presence of oxidant by measuring the decrease in absorption of green ABTS using a Thermo Multiskan Ascent Microplate Photometer at a wavelength of 734 nm, as compared with a Trolox (water soluble vitamin E analogue) standard. Since *Rhodococcus* biosurfactants are lipophilic, the samples of crude biosurfactants and isolated glycolipids were dissolved in 80% ethanol. The scavenging % of ABTS radicals was calculated using Eq. (1).

1 $$ \begin{gathered} ABTS~radical~scavenging~activity~\left( \% \right)~ \hfill \\ \quad = ~\frac{{Abs~\left( {control} \right) - ~Abs~\left( {sample} \right)}}{{Abs~\left( {control} \right)}} \times 100~ \hfill \\ \end{gathered} $$

### Gene induction and quantitative RT-PCR (RT-qPCR)

For *fbpB* gene expression under waste cooking oil, the *R. ruber* IEGM 231 strain was cultured in the abovementioned mineral RS medium containing 0.05 vol% of waste cooking oil or 1.0 g/L glucose (control). Cultures were grown to an approximate OD_600_ of 1.0 for 60 h before RNA extraction and purification using the RNA Solo kit (Evrogen, Russia). The cDNA synthesis was carried out with the MMLV RT kit (Evrogen, Russia). The qRT-PCR assay and data processing were performed using a CFX Connect™ Real-Time PCR System (Bio-Rad, USA) using the RT-qPCR Kit (Evrogen, Russia). Primers for *fbpB* gene were selected using the Primer-BLAST tool (NCBI, USA) as follows: forward 5’-TGGAGCCTGGTGTGACTCAT-3’, reverse 5’-GGCACCGATGGAGGACTTC-3’, and the length of the PCR-product of 998 bp. The enhancer for DNA synthesis from GC-rich matrix was used containing 0.54 M betaine, 1.34 mM dithiothreitol, 1.34% dimethyl sulfoxide, 11 mg/mL bovine serum albumin in water. The relative quantification of transcripts was calculated using the 2^−∆∆Ct^ method (Livak and Schmittgen 2001). All experiments were performed in triplicate, with the 16 S rRNA gene used as internal control.

### Statistics and multifactor analysis

Experimental data were statistically treated using a standard Excel program, calculating the mean and standard deviation (m ± SD) shown in figures as SD bars. The reliability of the differences in the average data was determined using the Student’s unpaired t-test.

Multifactor analysis was performed using Statistica 13.5.0.17 (TIBCO Software Inc.). The conditions were standardized as follows: the central and two boundary parameters were designated as levels 0, + 1 and − 1, respectively (Table 1); and all combinations of carbon and nitrogen source concentrations (27 in total) were indexed (Table S1). Pareto diagrams were constructed to show three factor’s effects and their statistical significance.

**Table 1:** Levels for carbon and nitrogen sources in multifactor analysis

Level	Vegetable oil concentration, vol%	Sugar concentration, g/L	KNO_3_/(NH_4_)_2_SO_4_ concentrations, g/L
+ 1	5.0	5.0	1.0/2.0
0	3.0	2.5	0.5/1.0
− 1	2.0	1.0	0.1/0.2

Response surface methodology (RSM) was used to determine the effect of three independent factors (concentrations of vegetable oil, sugar and N-salts) on the crude biosurfactant production (response factor). The optimal values of these independent factors were determined based on the obtained response surface graphs (response fitting).

The multifactor analysis was performed using the actual values of the factor concentrations. The codes + 1, 0 and − 1 were used solely as labels to designate the upper, central and lower boundaries of each factor range, and were not treated as equidistant numerical values in the regression model. The experimental design was a full three-level factorial (3³ = 27 runs), with all combinations of the three factor levels tested (Table S1). Response surface fitting was performed using the quadratic polynomial option. The Pareto chart was used for factor screening, while RSM was used for optimization of all factors jointly.

## Results and discussion

### Growth characteristics

**Fig. 1:**
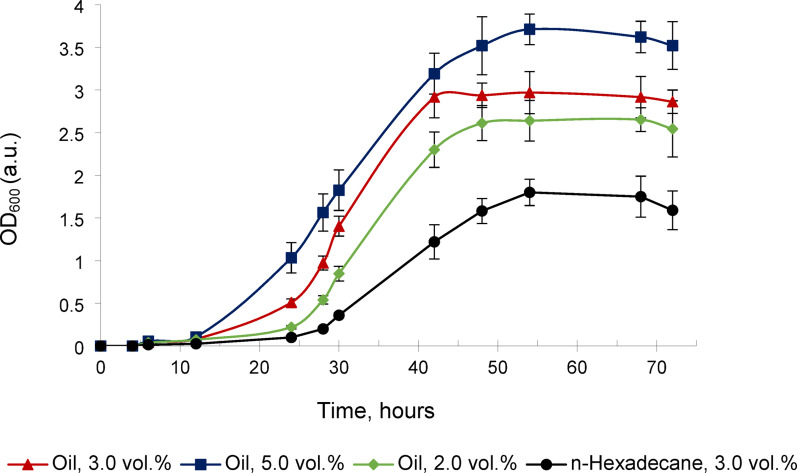
Comparative growth of *R. ruber* IEGM 231 using different concentrations of waste cooking oil. Means ± standard deviations for three independent replicates (*n* = 3) are shown

**Fig. 2:**
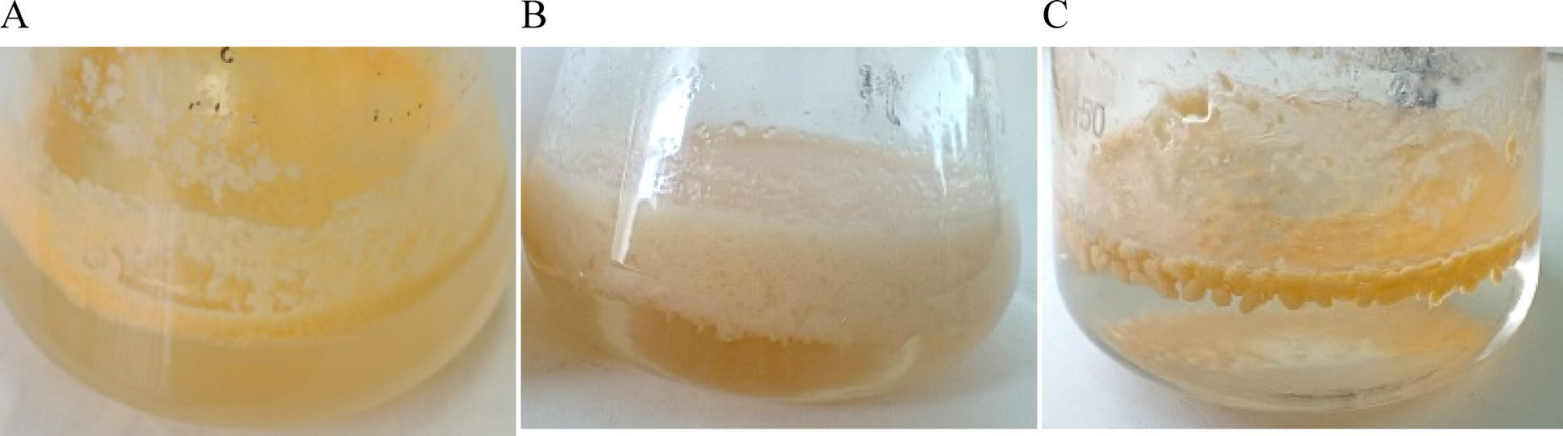
Morphological growth patterns of *R. ruber* IEGM 231 on waste cooking oil: **А**—standard floccular, typical for 2.0–3.0 vol% waste oil; **В**—slimy amorphous, characteristic for 5.0 vol% waste oil; **С**—globular, often recorded at 2.0 vol% waste oil

According to OD_600_ data (Fig. 1), the intensity of bacterial growth on waste cooking oil was 1.8 times higher than that when using the model substrate, *n*-hexadecane, at the same (3.0 vol%) concentration. The growth of the *R*. *ruber* IEGM 231 culture was proportional to the waste oil concentration. Different growth patterns were recorded at different concentrations of waste oil in the medium. In particular, three morphological groups were clearly distinguished (Fig. 2)—standard floccular, slimy amorphous, and globular structures. The main morphological form of culture was floccular, recorded in most experimental variants at 2.0–3.0 vol% waste oil. The slimy amorphous growth recorded at a high (5.0 vol%) waste oil concentration was presumably caused by incomplete assimilation of oil by rhodococcal cells. Globules 1–3 mm in size were found mainly when cells were grown in the presence of 2.0 vol% of oil, but not in all variants. Similar dense globules of different sizes were previously described for *Rhodococcus* growing under unfavorable conditions (Ivshina et al. 2021).

Thus, *R*. *ruber* cells used waste oil as the only carbon source and accumulated more biomass than growing on a hydrocarbon substrate (*n*-hexadecane). Several authors have concluded that waste cooking oil has an inhibitory effect on the growth of microbial cultures due to toxic compounds formed during frying, thus requiring its pretreatment with sorbents (Liepins et al. 2021). However, other studies did not reveal any negative effects of waste cooking oil on *Pseudomonas aeruginosa* or *Pseudozyma aphidis* and showed similar or even slightly higher oil consumption rate and biosurfactant production yield compared to refined oil (Chen et al. 2018; Niu et al. 2019).

### Extraction and structural elucidation of biosurfactants

The results on the production of biosurfactants using waste cooking oil and extracted with MTBE showed that largest amount (42.5 g/L) of biosurfactants was extracted from a culture grown with a maximum oil concentration (5.0 vol%), whereas with a minimum oil concentration (2.0 vol%), the yield of crude biosurfactants was only 18.0 g/L, and at an average oil concentration (3.0 vol%) it reached 24.3 g/L. Thus, the yield of crude biosurfactants was proportional to the concentration of waste cooking oil and exceeded the previously obtained yield of 10 g/L when using a model hydrocarbon substrate, *n*–hexadecane (Kuyukina et al. 2001; Philp et al. 2002). The extracted biosurfactants had a bright orange color and a viscous consistency.

**Fig. 3:**
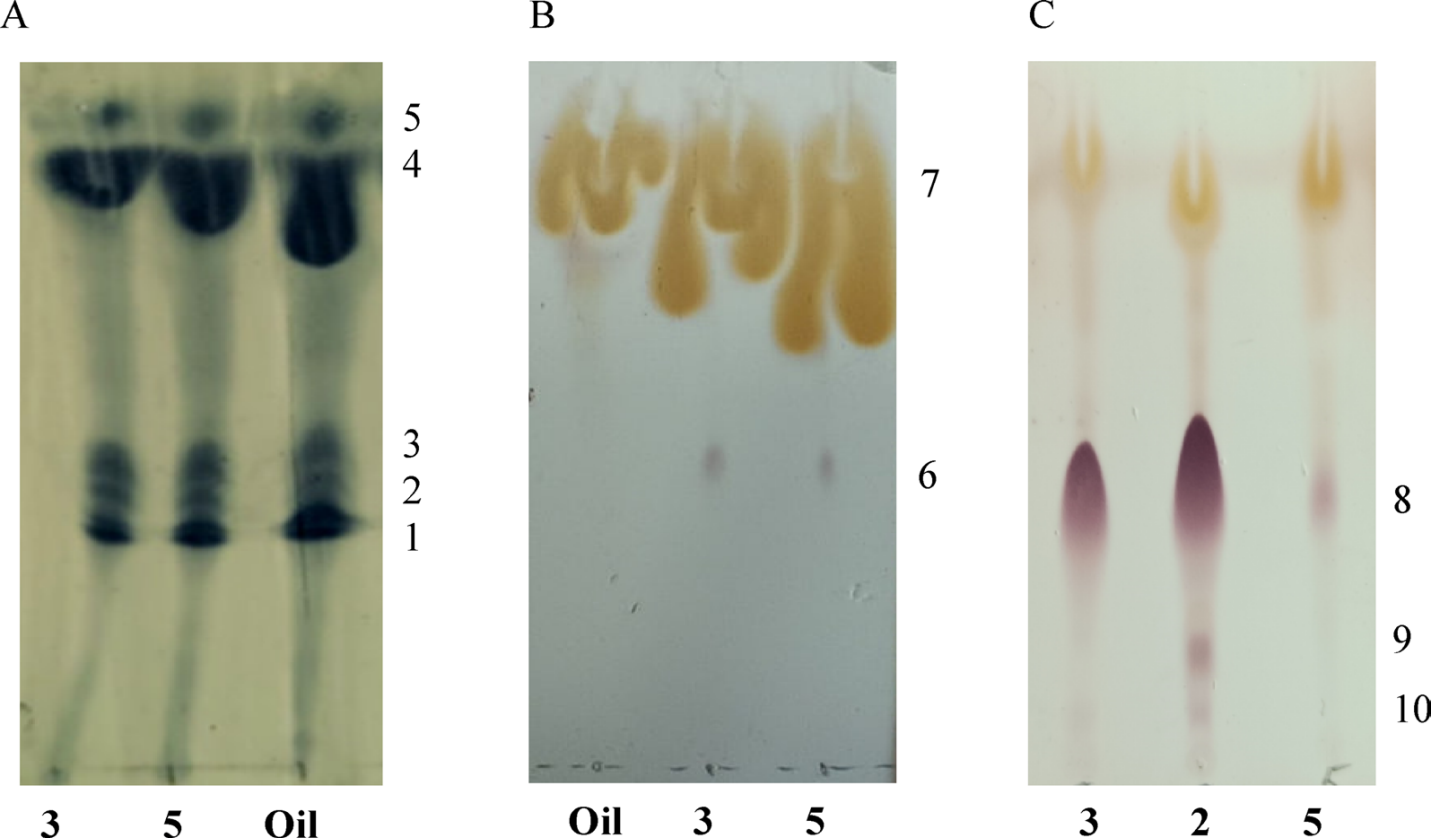
TLC separation of crude biosurfactant extracts (**A** and** B**) and glycolipid fractions isolated by flash chromatography (**C**). Solvent systems: A—*n*-hexane-chloroform-acetic acid (20:80:0.5) for total lipids; **B** and **C**—chloroform-methanol-water (85:15:2) for polar lipids. Spots were visualized with a 10% phosphoric-molybdenum acid in ethanol (**A**) and with 15% sulfuric acid (**B** and** C**) after heating to 110 °C. Experimental variants: Oil—waste cooking oil (control); 2, 3 and 5—extracts of biosurfactants when using 2.0 vol%, 3.0 vol% and 5.0 vol% of oil, respectively. Identified products: 1–monoacylglycerides; 2–diacylglycerides; 3–fatty acids; 4–triacylglycerides; 5–hydrocarbons; 6–glycolipids; 7–neutral lipids; 8–monoacyl trehalose; 9–diacyl trehalose; 10–trehalose dicorynomycolate

A qualitative analysis of extracted biosurfactants performed using TLC in a solvent system for total lipids (Fig. 3A) showed the presence of mono-, di-, and triacylglycerides (the latter in the largest amounts), as well as fatty acids and hydrocarbons. Compared with the initial sample of waste cooking oil, a relative decrease in the size of acylglyceride spots, especially the triacyl component, which are presumably transformed into glycolipids in rhodococcal cells, is clearly visible.

To confirm this assumption, crude biosurfactant extracts were further analyzed by TLC using a solvent system for polar lipids (Fig. 3B). It was noted that the glycolipid spot was present only in biosurfactant extracts and was absent in waste cooking oil (control), suggesting the transformation of acylglycerides into glycolipids. Based on the TLC results, it can be concluded from the size of the glycolipid spots that it is advantageous to use a lower concentration (3.0 vol%) of oil compared to 5.0 vol%.

By fractionating crude biosurfactant extracts using flash chromatography in a solvent system with increasing polarity, a glycolipid fraction was obtained (Fig. 3C), including its dominant component—monoacyl trehalose (Gein et al. 2020). Interestingly, the glycolipid fraction yield was inversely proportional to the concentration of the waste cooking oil and reached a maximum value (36.7 mg/L) with a minimum (2.0 vol%) oil concentration. At this oil concentration, three clearly distinguishable glycolipid spots can be seen on the chromatogram, presumably monoacyl trehalose, diacyl trehalose and trehalose dicorynomycolate, previously found in biosurfactants synthesized by this strain grown on *n*-hexadecane (Kuyukina et al. 2001; Philp et al. 2002).

### Optimization of biosurfactant production using RSM

Based on the results of the cultivation of *R. ruber* IEGM 231, with a three-fold variation of the three components of the nutrient medium, 27 samples of crude biosurfactant extracts were obtained, the concentrations of which are shown in Table 2. We have identified a direct strong correlation (0.95) between the yield of crude biosurfactants and the oil concentration used, with the largest average yield of 40.2 ± 4.9 g/L at the maximum oil concentration (5.0 vol%) and the lowest yield of 13.3 ± 1.2 g/L at the minimum (2.0 vol%) oil concentration. Thus, the degree of conversion of vegetable oil into biotechnologically valuable products—biosurfactants was 72–87%. However, the yield of crude biosurfactants obtained from a culture grown at a maximum oil concentration (5.0 vol%) showed a fairly wide range from 27.5 to 48.0 g/L, which can be explained by the assumption that higher concentrations of sugar and NH_4_- and NO_3_-containing salts had an inhibitory effect on bacterial assimilation of oil in excess. Interestingly, the crude biosurfactant yield in cultures characterized by slimy amorphous growth (see Fig. 2) was generally lower than in the cultures with standard floccular growth, thus suggesting incomplete oil assimilation by bacterial cells and its conversion into biosurfactants.

**Table 2:** A crude biosurfactant yield (g/L) depending on the varying parameters of the cultivation medium

Concentration of sugar, g/L	Concentrations of KNO_3_/(NH_4_)_2_SO_4_, g/L	Oil concentration, vol%		
		2.0	3.0	5.0
1.0	0.1/0.2	10.0 ± 1.3	14.7 ± 0.7	47.5 ± 3.5
1.0	0.5/1.0	14.7 ± 0.8	23.0 ± 1.1	48.0 ± 2.2
1.0	1.0/2.0	11.3 ± 0.5	22.7 ± 1.5	32.8 ± 5.5
2.5	0.1/0.2	13.7 ± 1.1	10.3 ± 1.2	40.0 ± 3.1
2.5	0.5/1.0	13.3± 0.6	22.0 ± 2.5	47.0 ± 3.8
2.5	1.0/2.0	13.7 ± 1.2	13.0 ± 1.3	33.5 ± 2.6
5.0	0.1/0.2	12.7 ± 0.4	13.3 ± 0.7	39.5 ± 2.9
5.0	0.5/1.0	14.7 ± 0.5	23.0 ± 1.4	27.5 ± 2.5
5.0	1.0/2.0	16.0 ± 0.4	15.7 ± 0.9	46.0 ± 3.7

Crude biosurfactant yield values represent means of three independent replicates (*n* = 3).

**Fig. 4:**
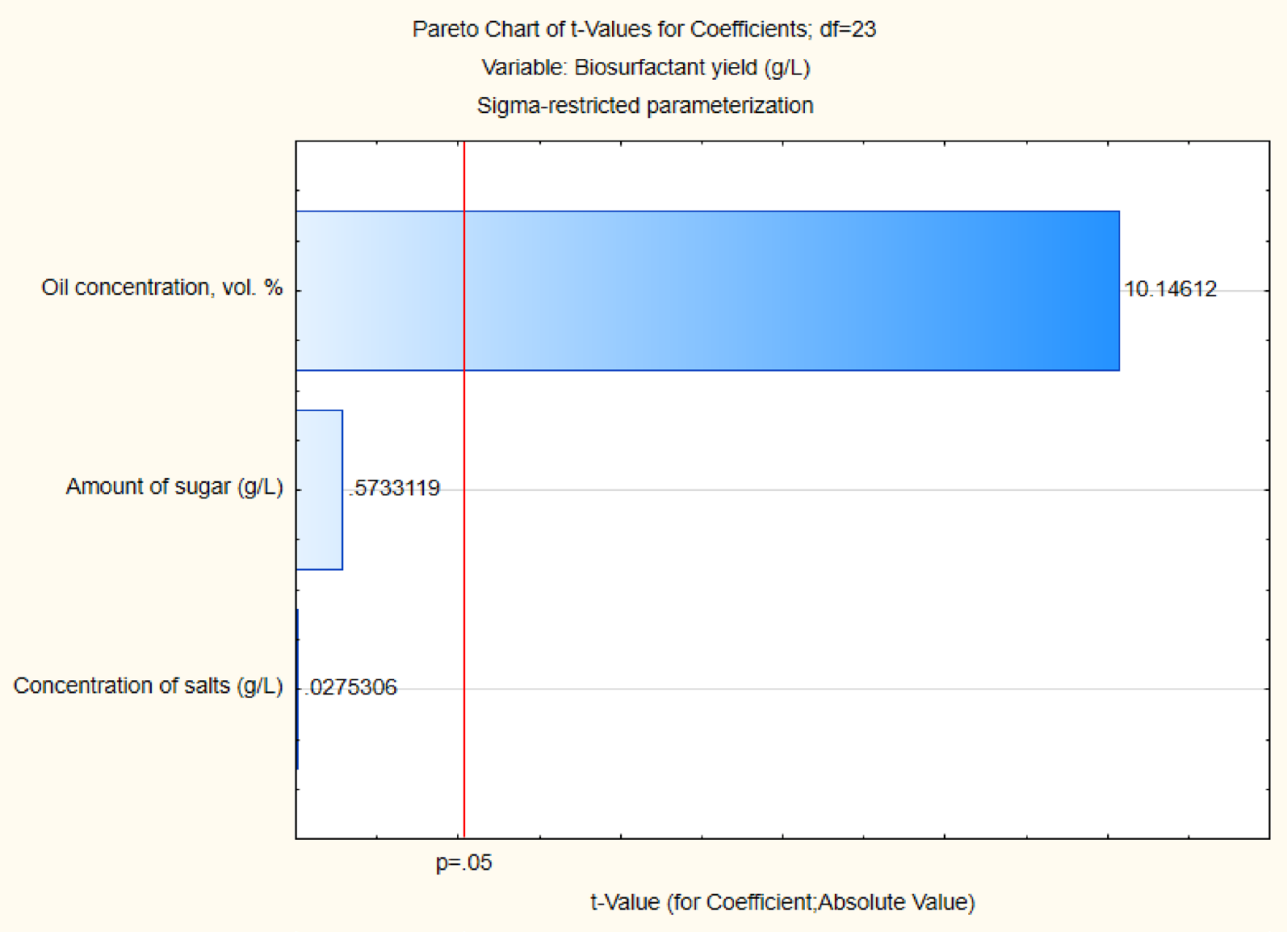
Pareto diagram of crude biosurfactant yields at varying parameters of the growth medium. Detailed statistics of the analysis is provided in Table S2, *n* = 27

Using standardized parameters, a Pareto diagram was constructed (Fig. 4), which shows a direct dependence of the crude biosurfactant yield on the oil concentration, and a statistically significant factor is also the oil concentration at a confidence level of *p* < 0.05. However, the concentration of sugar and nitrogen salts turned out to be insignificant factors, which required further research to select their optimal values.

**Fig. 5:**
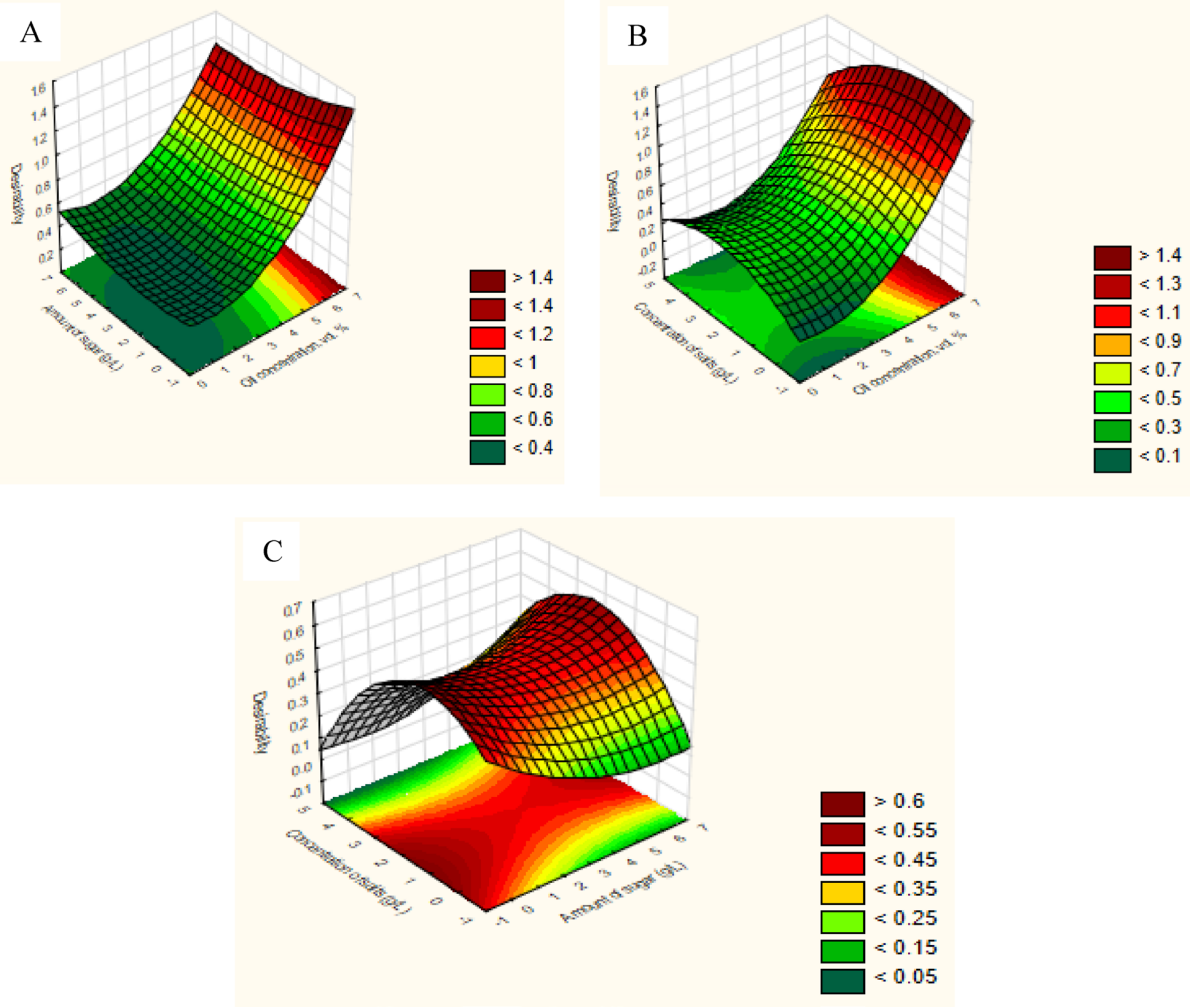
Response surface graphs (Desirability Surface; Method: Quadratic Fit) for crude biosurfactant yields at varying parameters of the growth medium. Detailed statistics of the analysis is provided in Table S3, *n* = 27

To determine the optimal combination of all three factors, a quadratic response surface model was subsequently fitted to the full dataset. Unlike the Pareto analysis, this model accounts for non-linear (quadratic) effects and two-way interactions between factors, enabling identification of the optimum within the experimental space. Using RSM, based on the obtained response surface graphs (Fig. 5), the optimal ratio of the parameters of the cultivation medium was determined for the maximum yield of crude biosurfactants: oil (+ 1), sugar (0), nitrogen salts (− 1) in the following concentrations: 5.0 vol%, 2.5 g/L, 0.1 and 0.2 g/L. Such low concentrations of nitrogen salts optimal for crude biosurfactant production correspond to previously obtained data on the positive effect of nitrogen limitation on the synthesis of biosurfactants by this strain (Philp et al. 2002) and other *Rhodococcus* species grown on hydrocarbon substrates (Ristau and Wagner 1983; Kim et al. 1990; Tuleva et al. 2008).

Interestingly, the amount of glycolipid fraction isolated from biosurfactants obtained under optimized growing conditions was inversely proportional to the oil concentration (27, 22, and 4.6 mg/L of glycolipids using 2.0, 3.0, and 5.0 vol% of oil), similar to the results obtained for waste cooking oil. This finding suggested that a significant part of the oil assimilated by *Rhodococcus* cells was present as neutral lipids (mainly acylglycerides) in crude biosurfactants, but was not converted into glycolipids.

### Emulsifying activity

The crude biosurfactant showed high emulsifying activity against petroleum hydrocarbons, food and cosmetic oils (Table 3). The formation of oil-in-water emulsions was recorded for most of the hydrophobic substrates studied, with the exception of the formation of reverse (water-in-oil) emulsions in the case of peach oil and jojoba oil. This property has biotechnological significance and indicates the possibility of using *Rhodococcus* biosurfactant as a stabilizer of lipophilic (oil based) emulsions widely used in cosmetology.

According to Table 3, the greatest emulsion index was observed for crude oil, which was 95% during the first minutes of emulsion formation and was maintained at 80% for 2 weeks. Slightly lower emulsion indices were found for kerosene and diesel fuel. These results confirm the possibility of the ecological use of *Rhodococcus* biosurfactant as an effective oil emulsifying and oil-washing agent in the treatment of oil-contaminated soils (Christofi and Ivshina 2001; Kuyukina and Ivshina 2019).

**Table 3:** Emulsifying activity (E, %) of crude biosurfactant from *R. ruber* IEGM 231

Substrates	Е_0.1,_ %	Е_1,_ %	Е_24,_ %	E_two weeks,_ %
*Petroleum products*				
*н*-Hexadecane	86.6 ± 3.5	66.7 ± 2.3	54.9 ± 1.7	ND
Kerosene	82.6 ± 8.0	76.4 ± 6.0	10.0 ± 2.3	8.0 ± 3.7
Diesel fuel	74.4 ± 3.3	75.2 ± 2.2	71.6 ± 4.4	71.2 ± 6.1
Mineral oil	67.0 ± 7.7	42.0 ± 4.1	22.7 ± 4.6	8.7 ± 6.8
Crude oil	95.0 ± 9.4	85.7 ± 7.1	83.2 ± 9.9	80.2 ± 9.4
*Food oils*				
Corn oil	70.4 ± 4.4	49.0 ± 9.8	38.7 ± 8.3	31.3 ± 11.8
Linseed oil	91.0 ± 8.8	75.2 ± 6.2	61.6 ± 4.4	28.0 ± 6.5
Olive oil	47.0 ± 8.2	43.0 ± 7.7	39.0 ± 5.0	30.7 ± 6.9
Sunflower oil	60.8 ± 8.2	60.0 ± 9.2	23.3 ± 4.7	17.3 ± 7.9
*Cosmetic oils*				
Jojoba oil	92.0 ± 9.6*	76.7 ± 8.6*	12.5 ± 3.4*	10.8 ± 5.4*
Peach oil	80.7 ± 8.6*	76.0 ± 7.4*	60.3 ± 9.3*	43.3 ± 8.4*

E_0.1_ is the emulsification index after 6 min, E_1_—after 1 h, E_24_—after 24 h, E_two weeks_—after 2 weeks. ND—no data, *water-in-oil emulsion. Means ± standard deviations for three independent replicates (*n* = 3) are shown

Among food and cosmetic oils, the highest emulsion indices (Е_0.1_ of 91–95%) were registered for linseed oil and jojoba oil, while the resulting emulsions were less stable than those obtained with hydrocarbon substrates, probably due to the presence of easily oxidizable polyunsaturated fatty acids, which can affect the stability of emulsions (Quezada et al. 2024). Thus, for most of oils, the emulsification indices gradually decreased to 10–30% within 2 weeks.

**Fig. 6:**
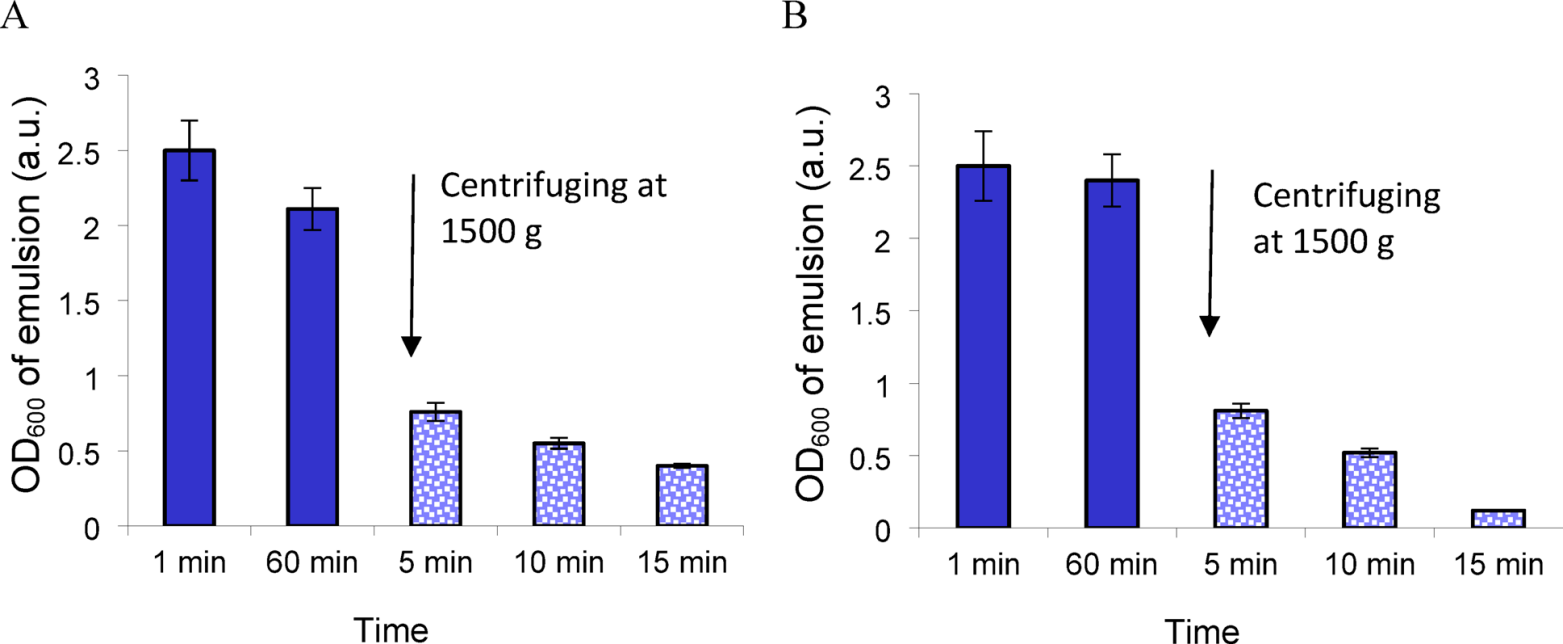
Hydrodynamic stability of emulsions formed with *n*-hexadecane (**A**) and sunflower oil (**B**). Means ± standard deviations for three independent replicates (*n* = 3) are shown

Rheological properties are crucial for emulsions used in various applications, such as cosmetic and food emulsions or metalworking liquids, because such products undergo shear during application. According to our data (Fig. 6), hydrodynamic stability values for the emulsions formed with hydrocarbons and oils were similar, and even after centrifugation at 1500 g for 15 min, these emulsions did not completely delaminate. In a recent study (Kłosowska-Chomiczewska et al. 2025), a crude rhamnolipid complex from *Pseudomonas* sp. SP-17 grown on glycerol showed better shear-thinning behavior of the resulting emulsions compared to purified rhamnolipids. Indeed, the emulsions produced with rapeseed oil using crude rhamnolipids were 1.5 times more resistant to shear stress (applied at a rotational speed from 2 to 400 s^− 1^) than those produced using commercial pure rhamnolipids. The authors concluded that crude and partially purified biosurfactants, due to this and other advantages such as cost-effectiveness, stability during long-term storage and biodegradability, can enter the market more effectively than more expensive pure products.

### Antioxidant capacity

**Fig. 7:**
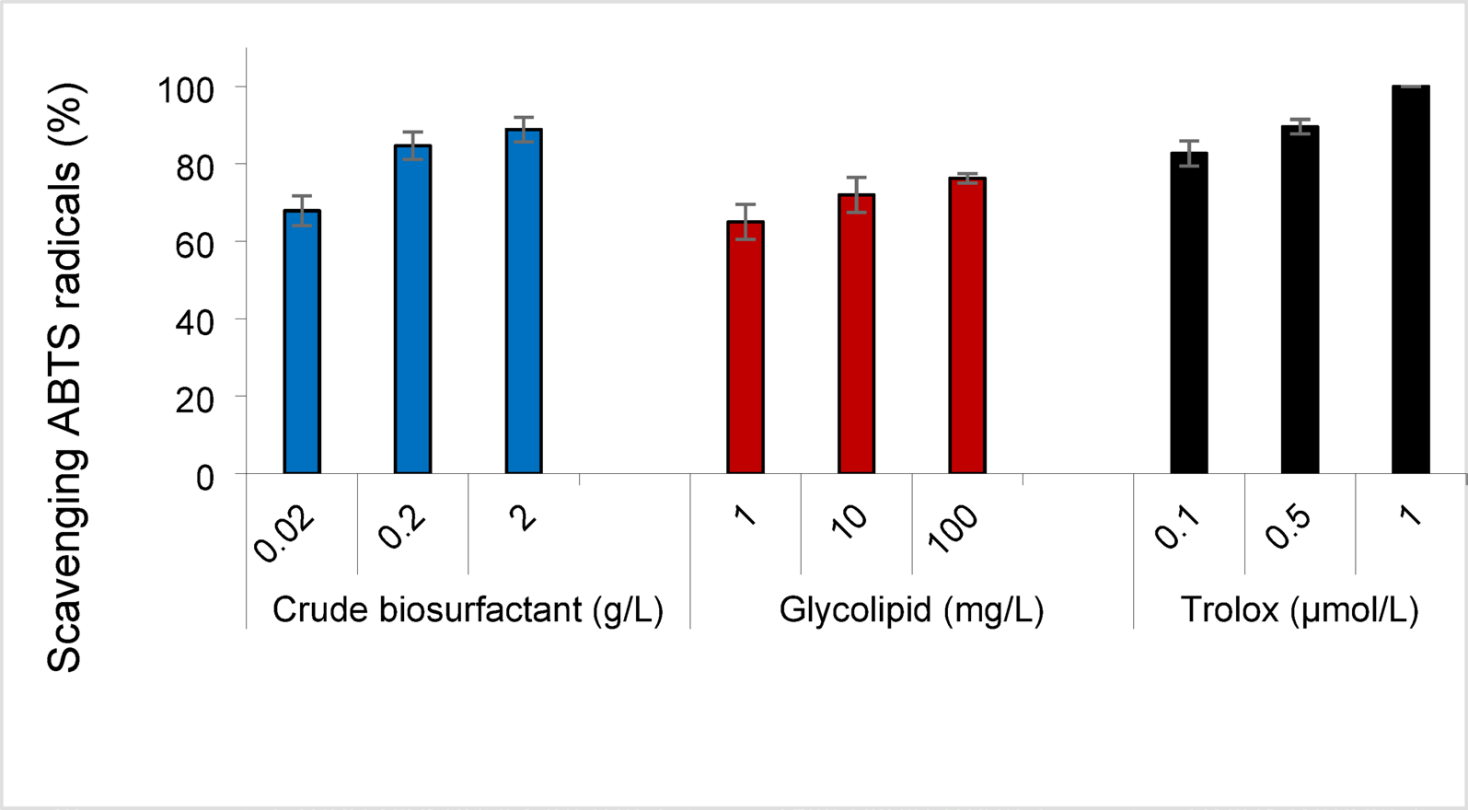
Antioxidant activity of biosurfactants at different concentrations. Means ± standard deviations for three independent replicates (*n* = 3) are shown

The antioxidant study of biosurfactants using the ABTS scavenging assay showed radical scavenging activity in a concentration-dependent manner (Fig. 7). Both the crude biosurfactant and the isolated glycolipid demonstrated high levels of antioxidant activity, comparable to Trolox (a vitamin E analogue). Interestingly, decimal dilutions of biosurfactants did not lead to a significant decrease in antioxidant activity, and even in the minimum concentrations tested (20 mg/L of crude biosurfactant and 1 mg/L of glycolipid), the corresponding 68 and 65% suppression of ABTS radicals was observed. Similarly, the biosurfactant produced by *Kocuria marina* (belonging to the same class *Actinomycetes*) using waste cooking oil showed maximum ABTS scavenging activity at the concentration of 1.5 g/L, comparable to butylated hydroxytoluene, a commonly used antioxidant (Brahma et al. 2024). However, when the concentration of biosurfactants from *K. marina* was reduced by 3 times (up to 0.5 g/L), its antioxidant activity decreased by half (up to 50% suppression of ABTS radicals). It should be noted that, despite the fact that the antioxidant activity of many microbial biosurfactants has been described in recent years, the molecular mechanisms underlying this action remain unknown (Tancredi et al. 2025). Presumably, the sugar moiety is responsible for antioxidant action, since trehalose, a disaccharide component of trehalolipids, is known to act as an antioxidant, protecting cells from damage caused by reactive oxygen species (Kuczyńska-Wiśnik et al. 2024). The predominant monoacyltrehalose component of *R*. *ruber* IEGM 231 glycolipid biosurfactants, containing one molecule of trehalose per only one fatty acid residue, is richer in trehalose than, for example, the well-known trehalose dimicolates from *Mycobacterium*, and, therefore, it is expected to have greater antioxidant activity. Crude biosurfactants from *R. ruber* IEGM 231 having high emulsifying and antioxidant activities can be used in food and cosmetic formulations. Whereas the purified glycolipid, which has shown antioxidant activity in this study in addition to the previously described immunoregulatory and anti-inflammatory activity (Gein et al. 2020; Kuyukina et al. 2020), may find biomedical applications.

### Expression of mycolyltransferase (*fbpB*) gene in the presence of waste cooking oil

**Fig. 8:**
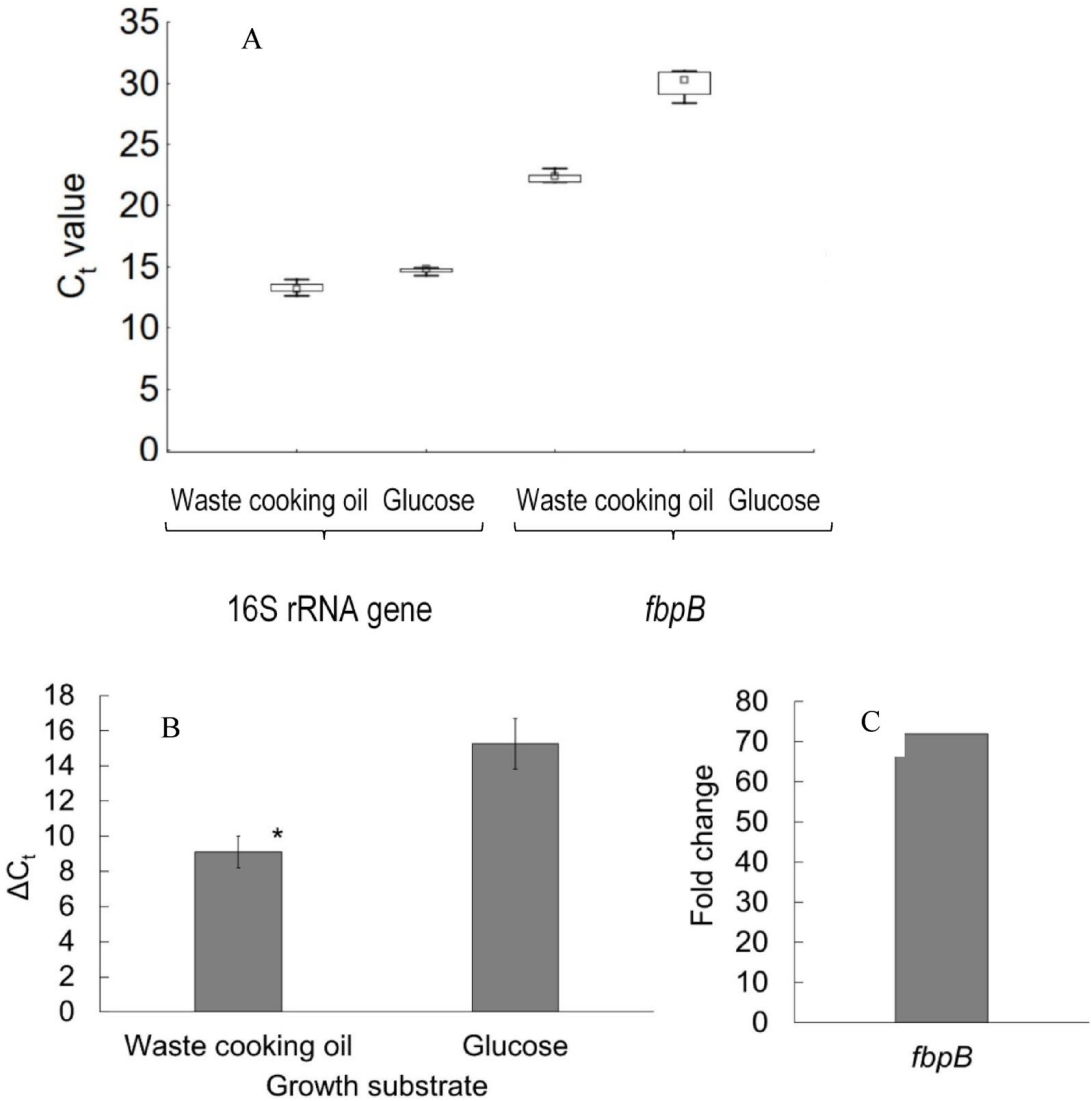
Relative expression levels of the *fbpB* gene in *R*. *ruber* IEGM 231 cells grown on waste cooking oil and glucose calculated using the 2–ΔΔCt method.** A**—Distribution of C_t_ values for the reference 16 S rRNA gene and the *fbpB* gene (points inside boxes depict medians, boxes indicate 25th and 75th percentiles, and the caps represent maximum and minimum values).** B**—The ΔCt values calculated for the *fbpB* gene relative to the reference gene (means ± standard deviations are shown; *statistically significantly differ from control (glucose) at *p* < 0.05; *n* = 3).** C**—A fold change of the *fbpB* gene expression in cells grown on waste cooking oil in comparison with glucose

To confirm the suitability of waste cooking oil for the synthesis of biosurfactants by *R*. *ruber* IEGM 231, we studied the expression of the *fbpB* gene encoding mycolyltransferase, a key enzyme of the final stage of trehalolipid assembly (Takayama et al. 2005). The *fbpB* transcript levels in *R*. *ruber* cells grown with waste cooking oil and glucose (used as a control) were quantified by RT-qPCR (Fig. 8). The relative expression level of the *fbpB* gene in cells grown on waste cooking oil was 72 times higher than when growing on glucose, which indicates the induction of the synthesis of glycolipid biosurfactants. The content of transcripts of this gene in cells growing on glucose is very low. This is evidenced by the high Ct value of 29.9 ± 1.2 (this is the threshold PCR cycle when the product begins to be detected—the higher this value is and closer to the 35th cycle, the lower the matrix content in the initial DNA/cDNA preparation). Whereas in the presence of oil, the amount of mRNAs synthesized from the *fbpB* gene increases.

Numerous experimental data demonstrate that rhodococcal strains exhibit pronounced substrate-dependent regulation of biosurfactant synthesis (Kuyukina and Ivshina 2019; Andreolli et al. 2023). Thus, on glucose and other hydrophilic substrates, the level of biosurfactant production did not exceed 0.2 g/L, while on hydrophobic substrates (*n*-alkanes, vegetable oils), the yield of biosurfactants increased by 5–100 times (Pirog et al. 2004; Franzetti et al. 2010). However, to the best of our knowledge, this is the first study to report the different expression of the biosurfactant synthesis gene depending on the substrate used. These findings provide new insights into the mechanisms of substrate-dependent regulation of biosurfactant synthesis in *Rhodococcus*.

## Conclusion

In this study, the *Rhodococcus ruber* IEGM 231 strain was able to grow well on vegetable oil, both native and post-frying, and synthesize biosurfactants in the growth-dependent mode with a yield 2–4 times higher than when grown on *n*-hexadecane, a model hydrocarbon substrate. Synthesized biosurfactants contained glycolipids with predominant monoacyl trehalose fraction similar to that described earlier for this strain grown on *n*-hexadecane. However, a considerable amount of residual acylglycerides from sunflower oil was detected in crude biosurfactants, thus indicating incomplete conversion of oil into glycolipids. To improve biosurfactant production, the growth medium, optimized using multifactor analysis and response surface method, contained sunflower oil, sugar, KNO_3_ and (NH_4_)_2_SO_4_ in the following concentrations 5.0 vol%, 2.5 g/L, 0.1 and 0.2 g/L, resulting in 40 g/L of crude biosurfactants. Such low concentrations of nitrogen salts optimal for crude biosurfactant production confirmed the positive effect of nitrogen limitation on the synthesis of biosurfactants by *Rhodococcus* when using a low-cost substrate, vegetable oil. It should be noted that growth conditions optimized in this study primarily contribute to the extraction of more crude biosurfactants suitable for environmental, food, or other industrial applications. However, conditions were also identified, in particular a lower oil concentration, which may be associated with a higher relative production of glycolipids. Furthermore, the revealed increased expression of the mycolyltransferase (*fbpB*) gene in *R. ruber* IEGM 231 cells grown in the presence of waste cooking oil opens the way to the genetic regulation of metabolic pathways involved in glycolipid synthesis. Further research may be aimed at elucidating the expression of other genes involved in the conversion of triacylglycerides from vegetable oil into glycolipid biosurfactants and their genetic engineering to develop cost-effective biotechnology. The biosurfactants synthesized on waste cooking oil showed a good emulsifying activity towards food and cosmetic oils, as well as high antioxidant potential, thus suggesting their possible applications in food industry and biomedicine.

## Supplementary Information

Below is the link to the electronic supplementary material.

Supplementary Material 1

## Data Availability

The dataset supporting the conclusions of this article is included within the article. Bacterial strain is available from the Regional Specialised Collection of Alkanotrophic Microorganisms (www.iegm.col).
