# Supplementary material for: Improvement of biosurfactant production by Rhodococcus ruber IEGM 231 grown on waste cooking oil, their emulsifying activity and antioxidant potential
Source: Bioresour Bioprocess. 2026 Aug 10;13(1):110. doi: 10.1186/s40643-026-01102-8 (PMC13457897; doi:10.1186/s40643-026-01102-8)
Supplement: Supplementary file 1 [file 40643_2026_1102_MOESM1_ESM.docx]

**Supplementary Information**

**Improvement of biosurfactant production by *Rhodococcus ruber* IEGM 231 grown on waste cooking oil, their emulsifying activity and antioxidant potential**

Maria S. Kuyukina^1,2*^, Anastasiia V. Krivoruchko^1,2^, Irina B. Ivshina^1,2^

^1^ Microbiology and Immunology Department, Perm State University, Perm, Russia

^2^ Institute of Ecology and Genetics of Microorganisms, Perm Federal Research Center, Perm, Russia

**Table S1.** Full 3³ factorial experimental design: factor levels and biosurfactant yields for all 27 runs.

| Run | Oil (vol. %) | Sugar (g/L) | KNO₃ (g/L) | (NH_4_)_2_SO₄ (g/L) | Biosurfactant yield (g/L) |
| --- | --- | --- | --- | --- | --- |
| 1 | 2.0 | 1.0 | 0.10 | 0.2 | 10.00 |
| 2 | 2.0 | 1.0 | 0.50 | 1.0 | 14.67 |
| 3 | 2.0 | 1.0 | 1.00 | 2.0 | 11.33 |
| 4 | 3.0 | 1.0 | 0.10 | 0.2 | 14.67 |
| 5 | 3.0 | 1.0 | 0.50 | 1.0 | 23.00 |
| 6 | 3.0 | 1.0 | 1.00 | 2.0 | 22.67 |
| 7 | 5.0 | 1.0 | 0.10 | 0.2 | 47.50 |
| 8 | 5.0 | 1.0 | 0.50 | 1.0 | 48.00 |
| 9 | 5.0 | 1.0 | 1.00 | 2.0 | 32.75 |
| 10 | 2.0 | 2.5 | 0.10 | 0.2 | 13.67 |
| 11 | 2.0 | 2.5 | 0.50 | 1.0 | 13.33 |
| 12 | 2.0 | 2.5 | 1.00 | 2.0 | 13.67 |
| 13 | 3.0 | 2.5 | 0.10 | 0.2 | 10.33 |
| 14 | 3.0 | 2.5 | 0.50 | 1.0 | 22.00 |
| 15 | 3.0 | 2.5 | 1.00 | 2.0 | 13.00 |
| 16 | 5.0 | 2.5 | 0.10 | 0.2 | 40.00 |
| 17 | 5.0 | 2.5 | 0.50 | 1.0 | 47.00 |
| 18 | 5.0 | 2.5 | 1.00 | 2.0 | 33.50 |
| 19 | 2.0 | 5.0 | 0.10 | 0.2 | 12.67 |
| 20 | 2.0 | 5.0 | 0.50 | 1.0 | 14.67 |
| 21 | 2.0 | 5.0 | 1.00 | 2.0 | 16.00 |
| 22 | 3.0 | 5.0 | 0.10 | 0.2 | 13.33 |
| 23 | 3.0 | 5.0 | 0.50 | 1.0 | 23.00 |
| 24 | 3.0 | 5.0 | 1.00 | 2.0 | 15.67 |
| 25 | 5.0 | 5.0 | 0.10 | 0.2 | 39.50 |
| 26 | 5.0 | 5.0 | 0.50 | 1.0 | 27.50 |
| 27 | 5.0 | 5.0 | 1.00 | 2.0 | 46.00 |

Biosurfactant yield values represent means of three independent replicates (n = 3).

**Table S2.** Linear multiple regression analysis underlying the Pareto chart (Figure 3). Regression coefficients, standardized coefficients (β), and significance tests for the effect of each factor on biosurfactant yield are shown

A. Regression coefficients

| Term | Coefficient | SE | Std. coefficient (β) | t-value | *p*-value | Significance |
| --- | --- | --- | --- | --- | --- | --- |
| Intercept | −6.218 | 4.149 | — | −1.499 | 0.1478 |  |
| Oil (vol. %) | 9.294 | 0.916 | 11.812 | 10.146 | <0.001 | *** |
| Sugar (g/L) | −0.397 | 0.692 | −0.668 | −0.573 | 0.572 |  |
| N-salts (g/L) | 0.085 | 3.103 | 0.032 | 0.028 | 0.9783 |  |

B. Analysis of variance (ANOVA)

| Source | SS | df | MS | F | *p-*value |
| --- | --- | --- | --- | --- | --- |
| Regression | 3638.23 | 3 | 1212.74 | 34.42 | <0.001 |
| Residual | 812.39 | 23 | 35.32 |  |  |
| Total | 4450.62 | 26 |  |  |  |

Model fit: R² = 0.818; Adjusted R² = 0.794; Root mean square error = 5.943; n = 27.

Analysis was performed with actual (non-coded) factor values using ordinary least squares. Standardized coefficient β indicates the change in biosurfactant yield (in SD units) per one SD change in the predictor. Significance codes: *** *p* < 0.001; ** *p* < 0.01; * *p* < 0.05; blank = not significant (*p* ≥ 0.05). SE – standard error, SS – sum of squares, df – degrees of freedom, MS – mean square.

**Table S3.** Quadratic response surface model (RSM) for biosurfactant yield, including regression coefficients, ANOVA, and predicted vs. observed values.

A. Quadratic model coefficients

Model equation: Ŷ = 12.339 − 4.658x₁ − 1.669x₂ + 22.626x₃ + 2.384x₁² + 0.355x₂² − 16.779x₃² − 0.573x₁x₂ − 2.729x₁x₃ + 1.866x₂x₃

| Term | Coefficient | SE | t-value | *p*-value | Significance |
| --- | --- | --- | --- | --- | --- |
| Intercept | 12.339 | 15.215 | 0.811 | 0.4283 |  |
| Oil (x₁) | −4.658 | 8.199 | −0.568 | 0.5774 |  |
| Sugar (x₂) | −1.669 | 4.135 | −0.403 | 0.6918 |  |
| N-salts (x₃) | 22.626 | 15.362 | 1.473 | 0.1586 |  |
| Oil² (x₁²) | 2.384 | 1.112 | 2.144 | 0.0470 | * |
| Sugar² (x₂²) | 0.355 | 0.588 | 0.604 | 0.5537 |  |
| N-salts² (x₃²) | −16.779 | 10.943 | −1.533 | 0.1436 |  |
| Oil × Sugar (x₁x₂) | −0.573 | 0.500 | −1.146 | 0.2679 |  |
| Oil × N salts (x₁x₃) | −2.729 | 2.242 | −1.217 | 0.2402 |  |
| Sugar × N salts (x₂x₃) | 1.866 | 1.695 | 1.101 | 0.2862 |  |

B. Analysis of variance (ANOVA)

| Source | SS | df | MS | F | *p*-value |
| --- | --- | --- | --- | --- | --- |
| Regression | 3963.44 | 9 | 440.38 | 15.39 | <0.001 |
| Residual | 486.59 | 17 | 28.62 |  |  |
| Total | 4450.03 | 26 |  |  |  |

Model fit: R² = 0.891; Adjusted R² = 0.833; RMSE = 5.350; n = 27.

C. Observed vs. predicted biosurfactant yields

| Run | Oil (vol. %) | Sugar (g/L) | N-salts (g/L) | Observed (g/L) | Predicted (g/L) | Residual (g/L) |
| --- | --- | --- | --- | --- | --- | --- |
| 1 | 2.0 | 1.0 | 0.10 | 10.00 | 11.84 | −1.84 |
| 2 | 2.0 | 1.0 | 0.50 | 14.67 | 15.42 | −0.75 |
| 3 | 2.0 | 1.0 | 1.00 | 11.33 | 12.36 | −1.03 |
| 4 | 3.0 | 1.0 | 0.10 | 14.67 | 18.25 | −3.58 |
| 5 | 3.0 | 1.0 | 0.50 | 23.00 | 20.75 | 2.25 |
| 6 | 3.0 | 1.0 | 1.00 | 22.67 | 16.32 | 6.35 |
| 7 | 5.0 | 1.0 | 0.10 | 47.50 | 45.40 | 2.10 |
| 8 | 5.0 | 1.0 | 0.50 | 48.00 | 45.71 | 2.29 |
| 9 | 5.0 | 1.0 | 1.00 | 32.75 | 38.55 | −5.80 |
| 10 | 2.0 | 2.5 | 0.10 | 13.67 | 9.76 | 3.91 |
| 11 | 2.0 | 2.5 | 0.50 | 13.33 | 14.47 | −1.14 |
| 12 | 2.0 | 2.5 | 1.00 | 13.67 | 12.80 | 0.87 |
| 13 | 3.0 | 2.5 | 0.10 | 10.33 | 15.32 | −4.99 |
| 14 | 3.0 | 2.5 | 0.50 | 22.00 | 18.93 | 3.07 |
| 15 | 3.0 | 2.5 | 1.00 | 13.00 | 15.90 | −2.90 |
| 16 | 5.0 | 2.5 | 0.10 | 40.00 | 40.74 | −0.74 |
| 17 | 5.0 | 2.5 | 0.50 | 47.00 | 42.17 | 4.83 |
| 18 | 5.0 | 2.5 | 1.00 | 33.50 | 36.41 | −2.91 |
| 19 | 2.0 | 5.0 | 0.10 | 12.67 | 9.85 | 2.82 |
| 20 | 2.0 | 5.0 | 0.50 | 14.67 | 16.43 | −1.76 |
| 21 | 2.0 | 5.0 | 1.00 | 16.00 | 17.09 | −1.09 |
| 22 | 3.0 | 5.0 | 0.10 | 13.33 | 13.98 | −0.65 |
| 23 | 3.0 | 5.0 | 0.50 | 23.00 | 19.46 | 3.54 |
| 24 | 3.0 | 5.0 | 1.00 | 15.67 | 18.76 | −3.09 |
| 25 | 5.0 | 5.0 | 0.10 | 39.50 | 36.53 | 2.97 |
| 26 | 5.0 | 5.0 | 0.50 | 27.50 | 39.83 | −12.33 |
| 27 | 5.0 | 5.0 | 1.00 | 46.00 | 36.40 | 9.60 |

Significance codes: * *p* < 0.05; blank = not significant (*p* ≥ 0.05). SE – standard error, SS – sum of squares, df – degrees of freedom, MS – mean square.
